# Review of Existing Models to Predict Reductions in Neural Tube Defects Due to Folic Acid Fortification and Model Results Using Data from Cameroon

**DOI:** 10.1093/advances/nmab083

**Published:** 2021-07-19

**Authors:** Hanqi Luo, Kenneth H Brown, Christine P Stewart, Laurel A Beckett, Adrienne Clermont, Stephen A Vosti, Jules M Guintang Assiene, Reina Engle-Stone

**Affiliations:** Department of Nutrition, University of California, Davis, CA, USA; Institute for Global Nutrition, University of California, Davis, CA, USA; Rollins School of Public Health, Emory University, Atlanta, GA, USA; Department of Nutrition, University of California, Davis, CA, USA; Institute for Global Nutrition, University of California, Davis, CA, USA; Department of Nutrition, University of California, Davis, CA, USA; Institute for Global Nutrition, University of California, Davis, CA, USA; Department of Public Health Sciences, University of California, Davis, CA, USA; Department of International Health, Johns Hopkins Bloomberg School of Public Health, Baltimore, MD, USA; Department of Agricultural and Resource Economics, University of California, Davis, CA, USA; Hellen Keller International, Yaoundé, Cameroon; Department of Nutrition, University of California, Davis, CA, USA; Institute for Global Nutrition, University of California, Davis, CA, USA

**Keywords:** neural tube defects, folic acid, fortification, Cameroon, nutritional models

## Abstract

Several models have been developed to predict the effects of folic acid fortification programs on prevention of neural tube defects (NTDs), but each relies on different assumptions and data inputs. We identified and reviewed 7 models that predict the effects of folic acid intake or status on NTD risk. We applied 4 of these models [the original and a modified version of the Lives Saved Tool (LiST) and models developed by Arth et al. and Wald et al.] to predict the effect of folic acid fortification of wheat flour on reduction of NTDs using national survey data from Cameroon. The estimated percentage of NTDs averted due to fortified wheat flour (5.0 μg folic acid/g flour) varied by predictive model, with a 21–31% reduction in LiST to 83% in Arth's model, and 15% in Wald's model. As the simulated fortification level was increased from 1.0 to 7.0 μg folic acid/g flour, the pattern of change in estimated numbers of NTDs averted differed due to different model assumptions: the number of NTDs averted increased and then reached a plateau in the modified LiST model (as would be expected in real-world conditions), increased sharply in Arth's model, and increased continuously in Wald's model. This wide variation in predicted effects, and implausible results in some cases, undermines the models’ utility for users of model outputs. Concurrent collection of dietary and biomarker data, including plasma and RBC folate concentrations, and NTD outcomes, is necessary to validate these models and monitor change in folic acid intake, folate-related biomarkers, and reduced NTD risk due to fortification. In the meantime, models based on erythrocyte folate concentration are recommended, based on biological plausibility and consistency with empirical evidence. Where erythrocyte folate data are unavailable, sensitivity analyses (using several models) could be conducted to examine the range of possible outcomes.

## Teaser

**Statement of Significance**: Inconsistent predictions by different models of neural tube defect (NTD) reduction due to folic acid fortification may cause confusion and hamper NTD prevention efforts; this paper offers suggested best practices for modeling.

## Introduction

Neural tube defects (NTDs) were estimated to affect 260,100 pregnancies worldwide in 2015 [95% uncertainty interval (UI): 213,800–322,000], with a global prevalence of 18.6 (15.3–23.0) per 10,000 live births (1). This number may be an underestimate, as it includes live births, stillbirths, and elective terminations of pregnancy for fetal anomalies (eTOPFA), but not NTD-related fetal losses in early pregnancy. Spina bifida and anencephaly, 2 of the most common types of NTDs, can be prevented by providing women with additional folic acid during the periconception period by means of supplementation or large-scale food-fortification programs (2, 3). Folic acid fortification programs may be more effective than supplementation programs for NTD reduction, because mandatory fortification provides folic acid to all consumers of the food vehicle without the need for additional behavior change, whereas periconception supplementation requires either a planned pregnancy or continuous supplementation of all women of reproductive age (WRA), successful social and behavioral change interventions to motivate adherence to supplementation, and a functional supply chain to deliver the supplements (4). Wheat flour fortification with folic acid has now been mandated or authorized in 83 countries and has been proven to reduce the prevalence of NTDs (5).

To encourage policymakers to implement a coherent and cost-effective national strategy for NTD prevention, policy advocates often attempt to estimate the number or proportion of NTDs and related deaths that could be prevented through policies such as food fortification with folic acid. Accordingly, several predictive models have been developed to quantify the relation between folic acid fortification and the number of NTDs averted (6–12). To convey a consistent policy message, it would be ideal if all these models yielded similar results; however, because the models use different data inputs, assumptions, and analytical methods, both the final estimates and the related policy implications (for example, which food vehicles and fortification levels to select) may differ. To our knowledge, there have been no efforts to compare these models systematically. Therefore, the current study was undertaken to *1*) review existing models to predict reduction in NTDs due to folic acid fortification, *2*) assess their degree of agreement based on input data from Cameroon, and *3*) compare the models in terms of the appropriateness of data inputs used and model assumptions, and the plausibility of their predicted changes in NTD prevalence with increasing fortification levels. This review and related analyses are intended to serve as a resource for health professionals, researchers, and policy advocates who may wish to apply these models or interpret the model results.

We used wheat flour fortification in Cameroon as a case study for several reasons. First, wheat flour is the food vehicle that was selected for mandatory folic acid fortification in Cameroon beginning in 2011 (13). Second, live-birth NTD prevalence in Cameroon was high prior to wheat flour fortification, at 19.9 per 10,000 live births in Yaoundé, the capital, which is ∼4 times the postfortification NTD prevalence in the United States (14), so generating estimates of NTDs averted is useful for policy discussions in Cameroon. Third, we had access to detailed information on dietary intake and micronutrient status through the 2009 Cameroon National Micronutrient Survey (CNMS), including national and subnational data on dietary folate and wheat flour intake (from 24-h dietary recalls) and plasma folate concentrations among women, which we could apply to the various models (15).

## Existing Models to Predict NTD Reduction Due to Folic Acid Intake

### Biological pathways underlying the relation between folic acid intake and NTD risk

**FIGURE 1:**
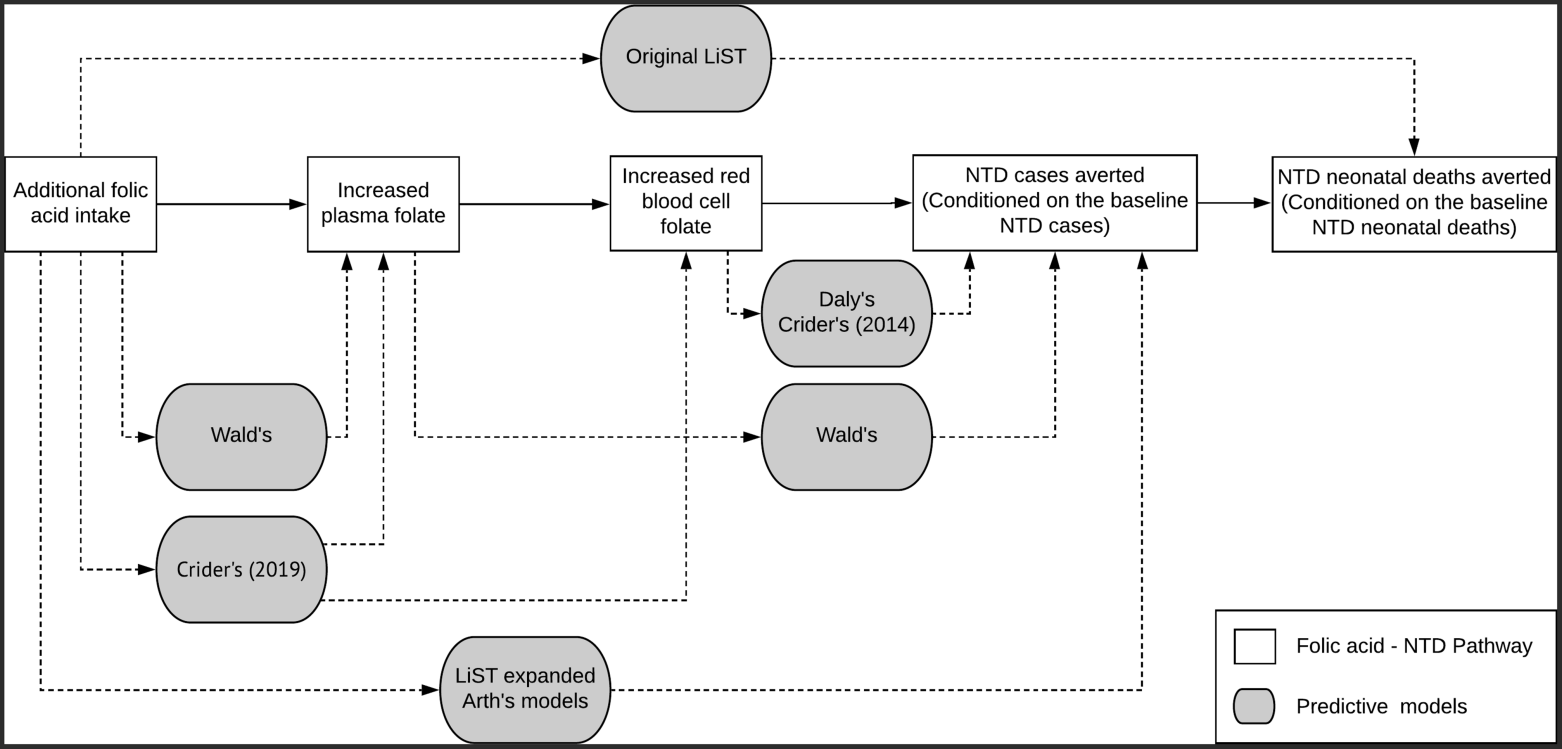
Biological pathway for the prevention of NTDs by folic acid fortification and relation to data inputs and outputs for folic acid–NTD-predictive models. LiST, Lives Saved Tool; NTD, neural tube defect.

To compare models that predict NTD risk reduction following folic acid–fortification programs, the first step is to understand the biological pathway linking folic acid intake and NTD reduction. These relations have been reviewed previously (10, 16) and are summarized in **Figure 1**. Briefly, additional folic acid intake leads to a relatively rapid increase in plasma folate concentration (11), and then, over a period of several months, to increased RBC folate concentration, which, among folate biomarkers, offers the closest approximation to embryo folate exposure (17, 18). Embryos with sufficient folate exposure in the 28 d following conception are less likely to develop NTDs (19). However, not all NTDs can be prevented by folic acid fortification because some NTDs may be caused by other environmental exposures and/or genetic factors. Most countries with mandatory food-fortification policies have achieved an NTD prevalence of 5.0–6.0 per 10,000 live births (20).

Evidence from both a nested case-control study in Ireland (9) and a recent modeling study (10) indicates that NTD prevalence decreases progressively as RBC folate concentration increases, reaching a stable level when the RBC folate concentration is >906 nmol/L (9, 10). Based on these observations, WHO recommends that the RBC folate concentration among WRA should be at least 906 nmol/L to minimize the risk of NTDs (18). There is some uncertainty regarding the exact amount of additional folic acid that should be consumed from fortified food or supplements to achieve specific RBC folate concentrations, particularly considering genetic variations in folate metabolism (21). Nevertheless, in settings where a fortified food is consumed by the majority of women, it is reasonable to assume that the prevalence of NTD will decrease in a dose-related manner as fortification levels (and, thus, folic acid intakes) increase, until reaching a threshold at which no further changes occur in the prevalence of NTDs with additional folic acid intake. Regarding the relation between plasma folate concentration risk (as opposed to RBC folate concentration) and NTD, there is no international consensus on the appropriate cutoff to minimize the risk of NTDs. (It has been widely recognized that serum and plasma folate have approximately the same values. In this paper, we use plasma folate to refer to both plasma and serum folate.) However, Chen et al. (12) recently proposed that a population-based plasma folate insufficiency threshold (pf-IT) of 25.5 nmol/L corresponds to the RBC folate insufficiency threshold of 906 nmol/L for optimal NTD risk reduction.

### Review of NTD-predictive models

We conducted 3 PubMed searches spanning the period January 1981–March 2021 using the keywords *1*) “folic acid” and “NTDs” (yielded 752 results), *2*) “folic acid” and “spina bifida” (yielded 562 results), or *3*) “folic acid” and “anencephaly” (yielded 351 results) to identify models that attempt to quantify the relation between maternal folate intake or status and NTD risk. We reviewed these search results and excluded articles that either did not present NTD prevention models or simply provided commentaries on or applications of previously existing models. We also conducted a thorough analysis of the NTD-related predictive models used in the Lives Saved Tool (LiST) (6). In the end, we identified 5 articles (7–11) that present original models to predict the relation between increased folic acid exposure via intervention programs, measured or estimated folic acid intake, or biomarkers of folate status and NTD risk (Figure 1). In addition, we included the default LiST method (6) and a modified version of the LiST method developed by our group but not previously published (Figure 1).

Two different modeling approaches are used in these papers to describe the relation between folic acid intake and NTD risk. In the first approach, the impact of folic acid fortification on NTDs is estimated based on the observed results from previous intervention trials or assumed program impact. We refer to this approach as a “direct estimate” of fortification program impact. Three models were developed using this approach: the default LiST model (6), which estimates NTD-affected neonatal deaths averted from folic acid fortification; a modified version of the LiST model adapted by our team to estimate all NTD cases averted; and a model developed by Arth et al. (7), which also estimates the number of NTD cases averted. The other approach, which we refer to as the “indirect approach,” estimates the effect of intervention on plasma or RBC folate concentration, and the subsequent effect of increased folate status on NTD risk. We identified 4 models that used the indirect approach: *1*) Daly's model (9), which is based on the relation between RBC folate concentration and NTD risk; *2*) Crider's 2014 model (10), which is based on the same relationship as for Daly's model; *3*) Wald's model (8), which estimates the effect of folic acid fortification on plasma folate concentration and the subsequent effect of increased plasma folate on NTD risk; and *4*) Crider's 2019 model (11), which estimates the effect of folic acid fortification/supplementation on plasma folate and RBC folate concentrations. As Crider et al. published 2 folic acid–NTD-predictive models (10, 11), we used the publication year to differentiate various models published by the Crider research group.

This indirect approach is particularly useful for planning fortification programs, because the model results can be used to estimate the effects of different fortification levels on folate biomarkers, which can, in turn, be compared with the respective thresholds for folate sufficiency [i.e., the 906-nmol/L RBC folate threshold (18) or the estimated pF-IT (12)]. Fortification program planners also can combine several models on the folic acid–NTD pathway to estimate NTD reduction. In the following section, we summarize the basic framework and assumptions of each of the direct and indirect models.

### Models using the direct approach to estimate the impact of folic acid–fortification programs

As noted above, 3 models provide a direct estimate of NTDs averted due to folic acid–fortification programs, namely the original and the modified LiST model (6) and a model developed by Arth et al. (7). These models are described in more detail below.

#### Original LiST model

The LiST model (6) was developed >10 y ago to estimate the effects of increased coverage of various health and nutrition interventions, including folic acid fortification and supplementation, on maternal and child mortality. The general approach taken by the LiST model is to first estimate the number of deaths attributable to various causes and then to estimate the proportion of those deaths that might be averted by scaling up the coverage of a specific intervention. The original LiST model estimates NTD-affected neonatal deaths (rather than all NTD cases) averted via folic acid fortification by multiplying NTD-related neonatal deaths at baseline by *1*) the change in coverage of folic acid interventions and *2*) a value representing the assumed effectiveness of folic acid for reducing NTD-affected neonatal deaths.

The original LiST model defines the change in folic acid program coverage as the change in “% of women [who are exposed to] appropriate fortification during pregnancy,” although “appropriate fortification” is not defined. The LiST model assumes that 100% program coverage can achieve an estimated effectiveness (% reduction in NTD-affected neonatal deaths) of 62% for supplementation programs (2), based on the results of 3 randomized controlled efficacy trials of folic acid supplementation, and 46% for fortification programs (2), based on the results of 8 population-based observational studies (**Supplemental Figure 1**). (The LiST model is frequently updated, and we assessed the model that was presented on their website at the time the analyses were completed.)

#### Modified LiST model

The original LiST model (6) has several limitations with regard to estimating changes in NTD risk that stem from its focus on neonatal deaths averted. Neonatal deaths are defined as infants who die in the first 28 d of life, but infants with NTDs, especially those with spina bifida, can survive longer than 28 d yet die later in childhood. Without specialized care, only ∼5% of children with spina bifida survive to 5 y of age in low- and middle-income countries (LMICs), although with optimal care, 53.3% of children with spina bifida can survive to 5 y of age (1). Therefore, the original LiST model underestimates the number of NTD-related deaths averted by only estimating the effect of fortification on NTD-affected neonatal deaths. Moreover, folic acid fortification could also prevent NTD-related stillbirths, which are not included in the original LiST model calculation. To address these limitations of the original LiST model, we created a modified version of the model by building a pathway between folic acid and NTD cases rather than neonatal deaths (**Supplemental Figure 2**). In this model, the baseline is the total number of NTD cases in the absence of folic acid fortification, which includes NTD-related stillbirths in addition to NTD-affected live births (14), with the assumption that infants with NTDs, especially those with spina bifida, can survive longer than 28 d yet die later in childhood (1). We calculated the NTD-related stillbirths based on the method published in 2018 by Blencowe et al. (1) by assuming the ratio of the prevalence of NTD-related stillbirths to the prevalence of NTD-affected live births in Cameroon is the same as that for all of sub-Saharan Africa, namely 2.067:1.00.

#### Arth's model

Arth's model (7) links women's folic acid intake and NTD cases averted through 3 model components: *1*) the annual number of potentially folic acid–preventable NTDs; *2*) the effectiveness of the fortification program, based on the amount of additional folic acid intake due to fortification; and *3*) program coverage (**Supplemental Figure 3**). [The original Arth's model (7) only estimated spina bifida and anencephaly that can be prevented by folic acid fortification. Even though there are other types of NTDs besides spina bifida and anencephaly, such as encephaloceles, for simplicity and consistency throughout the paper, we use NTD to refer to spina bifida and anencephaly, as used in Arth's model.]

The model developed by Arth et al. (7) first requires estimation of the number of NTD cases preventable by folic acid fortification assuming that the lowest NTD birth prevalence achievable following folic acid fortification is 5 per 10,000 live births, which is the NTD prevalence in the United States after ∼20 y of folic acid fortification (22). Thus, the difference in NTD prevalence between the country of interest and this lower threshold is used to compute the maximum proportion of NTD that can be prevented by folic acid fortification. The number of folic acid–preventable NTD cases is then calculated by multiplying the maximum proportion of preventable NTDs per 10,000 live births by the annual number of NTD-affected live births.

The Arth model then estimates the effectiveness of the fortification program based on the additional mean folic acid consumption. The population's mean additional folic acid consumption is estimated by multiplying the folic acid fortification level (micrograms per gram) by the average amount of the fortified product consumed by the population (grams). The model then assumes that if the population average folic acid consumption from fortified food is >150 μg/d, 100% of folic acid–preventable NTDs can be prevented; if the additional folic acid consumption falls between 20 to 150 μg/d, it is assumed that only 50% of folic acid–preventable NTDs can be prevented. The cutoff of 150 μg/d of additional folic acid from fortified cereals was based on the recommendation from the Teratology Society in 2014: “All governments institute mandatory folic acid fortification of a centrally produced food (such as, but not limited to, wheat flour, corn flour or meal, rice, and maize flour or meal) to provide almost all adults with at least an additional 150 μg of folic acid per day” (23).

Finally, program coverage was defined by Arth et al. (7) as “a country's flour that is produced in large-scale industrial mills (representing the flour that has the potential for fortification) × percentage of a country's industrial flour that is fortified with folic acid.” In their paper, the percentage of a country's industrial flour being fortified with folic acid was obtained from the Food Fortification Initiative's national database (24).

### Models using the indirect approach to estimate the effects of folic acid–fortification programs

As discussed above, additional folic acid intake from fortification leads to a rapid increase in plasma folate concentration, and then to increased RBC folate concentration over a period of several months, which can reduce women's risk of delivering an NTD-affected infant (10). The indirect approach generally models either the effect of folic acid fortification on RBC folate concentration or the effect of plasma or RBC folate concentrations on NTD risk. One exception is Wald's model (8), which considers the relations among folic acid intake, plasma folate concentration, and NTD risk.

#### Daly's model

In a landmark study published in 1995, Daly et al. (9) reported data from a nested case-control study that measured plasma and RBC folate concentrations and NTD outcomes among 56,049 women who gave birth in a hospital in Ireland (25). They presented the following logistic regression equation to describe the relation between RBC folate concentrations and NTD risk:

(1) $$\begin{eqnarray*}
\ln \left( {{\rm{odds\ of\ NTD\ risk}}} \right) = 1.6563 - 1.2193 \times \ln \left( {{\rm{RBC}}} \right)
\end{eqnarray*}$$

Based on this equation, Daly et al. concludes that, among women with RBC concentration <340 nmol/L, the risk of delivering an NTD-affected infant is 66 in 10,000. With greater RBC folate concentration, the prevalence of NTD risk decreases progressively, reaching a nadir when RBC folate concentrations are >906 nmol/L.

#### Crider's 2014 model

Crider et al. (10) used a Bayesian approach to study the association between a mother's RBC folate concentration in early pregnancy and the risk of delivering an NTD-affected infant, using data from 2 trials: *1*) the Community Intervention Project in 2 regions of China (Hebei province in the north and Zhejiang and Jiangsu provinces in the south) (26) and *2*) the Folic Acid Dosing Trial conducted in northern China (Hebei Province) (27). Crider et al. developed a logistic regression model to assess the association between RBC folate and NTD risk, as follows:

(2) $$\begin{eqnarray*}
\ln \left( {{\rm{odds\ of\ NTD\ risk}}} \right) = {\rm{\ }}4.57 - 1.70 \times {\rm{ln}}\left( {{\rm{RBC}}} \right)
\end{eqnarray*}$$

Crider's 2014 model also concludes that the risk of NTDs is attenuated at RBC folate concentrations >1000 nmol/L, which is consistent with the results published by Daly et al. (9). Crider's 2014 model is particularly advantageous because it also incorporates the effect of methylene tetrahydrofolate reductase (MTHFR) 677 genotype on determining how RBC folate responds to folic acid supplementation and fortification.

#### Wald's model

Wald et al. (8) modeled a dose–response relation among folic acid intake, plasma folate concentration, and NTD risk. Based on data from 13 folic acid supplementation trials conducted between 1965 to 2000, they described a linear relation between additional folic acid intake from fortification programs and the change in plasma folate concentration, as indicated by the following equation:

(3) $$\begin{eqnarray*}
&&{\rm{Predicted\ plasma\ folate}}\nonumber\\
&&\quad = {\rm{original\ plasma\ folate}}+ 0.0227\nonumber\\
&&\qquad \times\, {\rm{additional\ folic\ acid\ intake\ }}
\end{eqnarray*}$$

[In the original paper of Wald's model (8), the unit of plasma folate concentration is ng/mL. To be consistent with the rest of this paper, we converted the equation so that the unit of plasma folate is nmol/L.] They also assessed the relation between change in plasma folate concentration and change in NTD risk (Eq. 2), using the Irish case-control study published by Daly et al. (9), and found a log-log relation between the plasma folate concentration and NTD risks. Wald et al. re-wrote this log-log regression and derived an equation to estimate the % reduction in NTDs, as shown in the following equation:

(4) $$\begin{eqnarray*}
&&{\rm{\% \ NTD\ reduction\ }}\nonumber\\
&&\quad = \left( {1 - {\rm{\ }}{{\left( {\frac{{{\rm{new\ or\ predicted\ plasma\ folate\ }}}}{{{\rm{original\ plasma\ folate\ }}}}} \right)}^{ - 0.81}}} \right){\rm{\ }}\nonumber\\
&&\qquad \times 100
\end{eqnarray*}$$

#### Crider's 2019 model

Crider et al. (11) carried out a systematic review of the effect of folic acid intervention trials on plasma and RBC folate concentrations. They identified 97 trials that reported change in plasma folate concentration and found that the ratio of steady-state to baseline plasma folate concentration increases by 11.6% for each 100-μg/d increment in additional folic acid intake. Their findings are summarized in the equation below (Eq.   5):

(5) $$\begin{eqnarray*}
&&{\rm{Predicted\ serum\ folate}}\nonumber\\
&&\quad= {\rm{baseline\ serum\ folate}}\nonumber\\
&&\qquad \times\, {1.116^{\left( {{\rm{folic\ acid\ intake\ }}\left[ {{\rm{\mu g}}} \right]/100} \right)}}
\end{eqnarray*}$$

Crider also found 23 articles that describe changes in RBC folate following folic acid intervention. Because of the limited range of folic acid intake in the studies that were identified, they could only report on RBC concentration responses to additional folic acid intake in the range between 375 μg/d and 570 μg/d. Within this range of additional folic acid intake, RBC folate concentrations increase 78% from baseline to steady state over a median of 36 wk of increased folic acid intake.

## Application of Models to the Case of Cameroon

Our second objective was to compare the outputs of the various models when applied to data from the same test case. For these analyses, we used 24-h dietary intake data from the women in the 2009 CNMS (*n* = 902) (13). The details of this study have been published previously (13). In brief, the CNMS was a nationally representative, multistage, cluster survey, which provided information on the population's food and micronutrient intakes and micronutrient status. Women's (*n* = 902) dietary intakes were assessed using the 24-h dietary recall method (with replicates in a 10% subset of participants) based on an assessment tool specifically developed for use in low-income African populations with low literacy rates (28). Venous blood specimens were collected by trained phlebotomists into tubes containing lithium heparin as an anticoagulant (Sarstedt). Plasma was separated by centrifugation and frozen on the day of collection at < −20°C. Plasma vials were wrapped in aluminum foil to avoid exposure to light. Analysis of folate in plasma samples was carried out at the Western Human Nutrition Research Center in Davis, California, using the SimulTRAC-SNB Radioassay Vitamin B-12 [57Co]/Folate[125I] Kit (MP Biomedicals) (15).

First, we compared the 3 direct models that use estimates of exposure to additional folic acid intake from fortification programs to predict NTDs averted—namely, the original and modified LiST models (6) and the model developed by Arth et al. (7). Because Wald's model also can predict NTDs averted from additional folic acid intake through its effect on plasma folate concentration (8), we included the results from Wald's model for comparison with the 3 direct approach models. Then, we compared 2 indirect approach models: Wald's (8) and Crider's 2019 (11) models that use estimates of folic acid intake from a fortification program to predict plasma folate concentration change. As noted above, we were unable to include models published by Daly et al. (9) and Crider et al. (2014) (10), which require information on RBC folate concentration prior to fortification as a model input, because RBC folate was not measured in the CNMS.

For all of these analyses, we used a common set of baseline population characteristics to facilitate comparison of results (**Table 1**). Based on the available data, baseline NTD-affected neonatal deaths (as used by the original LiST model) and NTD cases (i.e., NTD-affected birth outcomes, which were used in all other models) were calculated. Specifically, the baseline number of NTD-affected neonatal deaths in Cameroon was calculated in the original LiST model by multiplying *1*) the percentage of neonatal deaths from congenital anomalies that are attributable to NTDs by *2*) the total number of neonatal deaths due to congenital anomalies. Blencowe and colleagues (4) estimated that, in 2010, 16.7% (UI: 6.7–66.7%) of neonatal deaths from congenital anomalies were due to NTDs in central Africa, and we applied this same estimate for Cameroon. The total number of neonatal deaths due to congenital anomalies was estimated to be 1487, based on the data from the United Nations Interagency Group for Child Mortality (29). Hence, the baseline NTD-affected neonatal deaths is 16.7% × 1487 = 248.

**TABLE 1:** Model inputs used to compare existing models of the effect of folic acid fortification on risk of NTDs: estimates of NTD prevalence, folic acid intake, and folate status among women of reproductive age for Cameroon (2009)^1^

Model inputs (reference)	Estimates
Baseline NTD-affected neonatal deaths	
Number of neonatal deaths due to congenital anomalies (29)	1487
Percentage of neonatal deaths due to congenital anomalies that are attributed to NTDs (4)	16.7
Number of NTD-affected neonatal deaths^2^	248
Baseline NTD cases	
Number of live births (30)	790,346
NTD-related eTOPFA per 10,000 live births^3^	0
NTD-affected live births per 10,000 live births (14)	19.9
NTD-related stillbirth prevalence per 10,000 live births	9.6
Overall envelope prevalence of NTDs per 10,000 live births^4^	29.5
Number of NTD cases^5^	2334
Folic acid intake from fortified wheat flour	
National usual wheat flour consumption, g/d	36.4
Usual wheat flour consumption in 2 major cities, g/d	46.3
National average additional folic acid intake at the target fortification level,^6^ μg/d	182.0
Population average additional folic acid intake at the target fortification level in 2 major cities,^6^ μg/d	231.5
Reach (13),^7^ %	45.9
Percentage reduction in inadequate intake,^8^ %	50.1
Folate status, nmol/L	
Baseline plasma folate concentration (15)	18.0
Baseline plasma folate concentration in 2 major cities (31)	14.8
Plasma folate concentration after 1 y of folic acid fortification in 2 major cities (31)	46.9

1 eTOPFA, elective termination of pregnancy for fetal anomalies; NTD, neutral tube defect.

2 The baseline number of NTD-affected neonatal deaths in Cameroon was calculated by multiplying the percentage of neonatal deaths from congenital anomalies that are attributable to NTDs (16.7%) by the total number of neonatal deaths due to congenital anomalies (1487).

3 We assumed that the prevalence of eTOPFA is 0, as prenatal screening is not widely available, and elective termination is illegal in Cameroon.

4 The overall envelope of prevalence of NTDs is calculated as the sum of NTD-related eTOPFA, stillbirths, and NTD-affected live births divided by the total number of live births (1).

5 The baseline number of NTD cases was estimated by multiplying the overall envelope prevalence of NTDs (29.5 per 10,000 live births) by the number of live births (790,346). Note: the numbers may not add up because of rounding.

6 The population average additional folic acid intake is estimated by multiplying the folic acid fortification level by the mean usual wheat flour consumption. The target folic acid fortification level is 5 μg folic acid per gram of wheat flour.

7 Reach is defined as percentage of women consuming fortified wheat flour.

8 Percentage reduction in inadequate intake is defined as percentage of the total population who have shifted from inadequate to adequate intakes following introduction of fortified wheat flour.

The baseline number of NTD cases was estimated by multiplying *1*) the overall “envelope” prevalence of NTDs by *2*) the number of live births. The overall “envelope” prevalence of NTDs was defined by Blencowe and colleagues (2018) (1) as the sum of NTD prevalence among live births, stillbirths, and eTOPFA. We assumed that the NTD prevalence at the national level in Cameroon is equal to the prevalence observed in 3 main hospitals in Yaoundé between 1997 and 2006, which is 19.9 NTDs per 10,000 live births (14); that the ratio of NTD-affected live births to NTD-related stillbirths is the same in Cameroon as reported for all of sub-Saharan Africa (1), which is 2.067:1; and that the prevalence of eTOPFA is zero, as prenatal screening is not widely available and elective termination of pregnancy is illegal in Cameroon. The NTD-related stillbirths are then calculated as 19.9/2.067= 9.6 per 10,000 live births. Therefore, the overall “envelope” is (19.9 live births) + (9.6 stillbirths) + (0 eTOPFA) = 29.5 NTDs per 10,000 live births. The number of live births in Cameroon is 790,346, based on the United Nations World Population Prospects national projections, provided by the LiST (30). Hence, the number of NTD cases equals (790,346 live births) × (29.6/10,000 NTD prevalence) = 2334. (The numbers may differ slightly because of rounding.)

### Application of models

We applied data from Cameroon to the various models, assuming that 100% of wheat flour was fortified with 5 μg folic acid per gram of wheat flour, which is the level of fortification mandated in Cameroon. A detailed description of the methods for applying each of the models to the available data from Cameroon is presented in **Supplemental Methods 1**.

### Comparison of models at the target fortification level

For the percentage of NTD-affected neonatal deaths or NTD cases averted, the original LiST (6) and modified models yielded the same prediction for estimate #1 (∼21%, based on reach), and predictions for estimate #2 were similar to each other (∼31% and ∼29%, respectively, based on effective coverage) (**Table 2**). The estimated number of NTDs averted according to the modified LiST model was substantially higher than that estimated by the original LiST model (493 vs. 52 for estimate #1 and 724 vs. 71 for estimate #2), because the modified LiST model focuses on all NTD cases, rather than just neonatal deaths, and includes stillbirths and cases surviving past the neonatal period. When predicting the number of NTD cases averted, the modified LiST (6), Wald's (8), and Arth's (7) models generated substantially different results, ranging from 359 cases averted (Wald's model) to 1938 cases averted (Arth's model) annually, representing ∼15% to 83% of total NTD cases, respectively.

**TABLE 2:** Comparison of the number and percentage of NTDs averted by the current wheat flour fortification program (5.0 μg folic acid/g) in Cameroon, as estimated by the LiST original and modified models and Arth's, and Wald's predictive models^1^

Method	Total annual number of NTD neonatal deaths or NTD cases (in absence of fortification)	Number of NTDs averted annually	Percentage of NTDs averted annually
Original LiST estimate #1 (lower bound) (6)	248	52	21.1
Original LiST estimate #2 (upper bound) (6)	248	71	28.6
Modified LiST estimate #1	2334	493	21.1
Modified LiST estimate #2	2334	724	31.0
Arth et al. (7)	2334	1938	83.1
Wald et al. (8)	2334	359	15.4

1 LiST, Lives Saved Tool; NTD, neural tube defect.

The 2 indirect models, Wald's (8) and Crider's 2019 (11) models, generated similar predictions of the postfortification plasma folate concentrations in 2 major cities in Cameroon: 20.1 and 19.1 nmol/L, respectively. Both Wald's and Crider's 2019 models predicted that plasma folate concentrations would remain below the pf-IT suggested by Chen et al. (12) (25.5 nmol/L). In reality, after 1 y of wheat flour fortification, the observed mean plasma folate concentration among women in the same area was 46.9 nmol/L (31), which is far above the pf-IT. Thus, although Wald's and Crider's 2019 models yielded similar results, both models underestimated the observed change in plasma folate concentrations in 2 major cities in Cameroon.

### Dose–response relation

**FIGURE 2:**
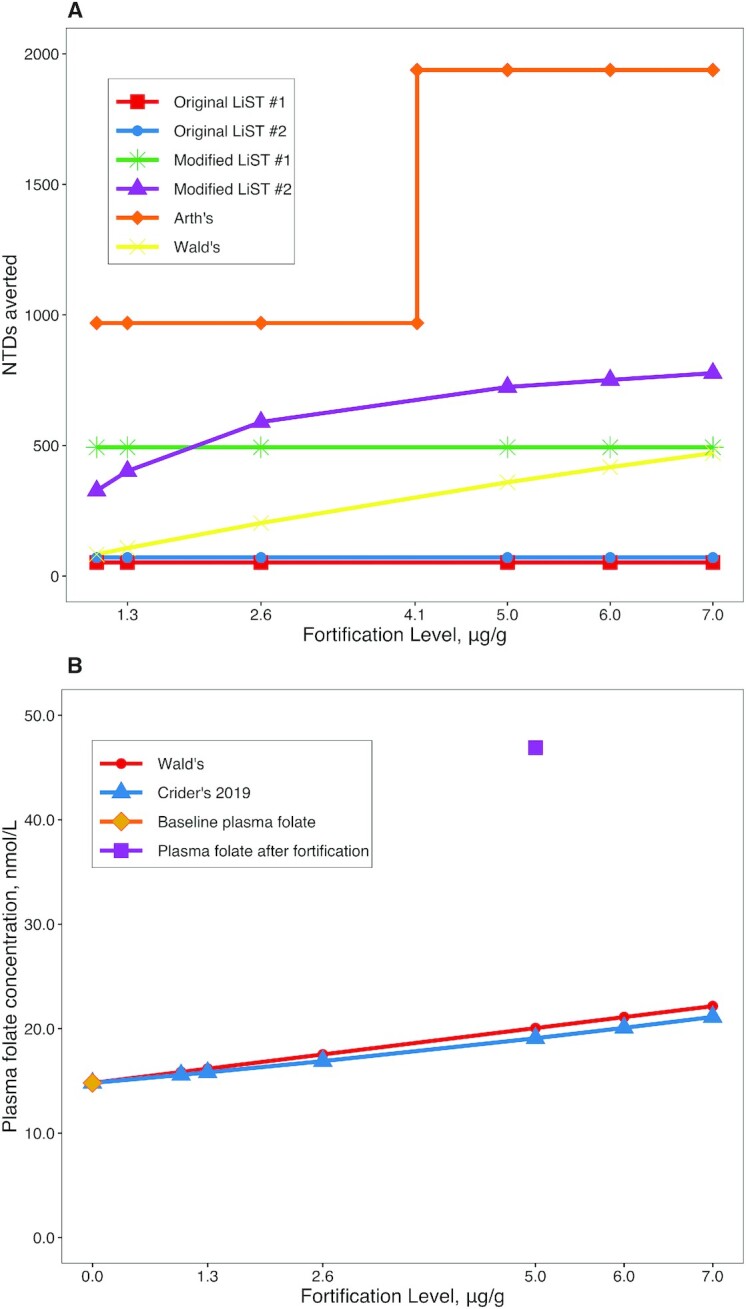
Comparison of the predicted effect of various fortification levels of wheat flour on (A) the number of NTDs averted as estimated by the LiST original and modified models and Arth's and Wald's predictive models and (B) the predicted plasma folate concentrations in 2 major cities of Cameroon, as estimated by Wald's and Crider's 2019 models. The LiST original model predicts the number of NTD-affected neonatal deaths averted, whereas other models predict the number of NTD cases averted. LiST, Lives Saved Tool; NTD, neural tube defect.

We also estimated how different levels of folic acid fortification would influence the predicted number of NTDs averted (**Figure 2**A) or the predicted plasma folate concentration (Figure 2B) by simulating fortification levels of 1.0, 1.3, 2.6, 5.0, 6.0, and 7.0 μg/g. Fortification levels of 1.0, 1.3, 2.6, and 5.0 μg/g are based on the WHO guidelines for wheat and maize flour fortification, depending on the average wheat flour consumption per capita [>300, 150–300, 75–149, and <75 g/d, respectively (32)]. Fortification levels of 6.0 and 7.0 μg/g were used to assess whether even higher fortification levels could further reduce NTDs according to these models.

As illustrated in Figure 2A, estimates derived from both the original LiST model (6) and estimate #1 of the LIST modified model suggested a constant number of NTD-affected neonatal deaths averted, regardless of the level of folic acid fortification, because the reach metric does not consider the amount of folic acid provided. In contrast, based on the LiST estimate #2 (% reduction in inadequate intake × supplementation effectiveness) of the modified LiST model, the predicted number of NTD deaths/cases averted increased as the fortification levels increased and then flattened out at a fortification level of 5 μg/g. Arth's model (7) predicted that the number of NTD cases averted would double, from 969 to 1938, when the wheat flour fortification level increased from 2.6 μg/g to 5.0 μg/g (i.e., when mean additional folic acid intake surpassed the 150-μg/d threshold). Based on the mean usual wheat flour consumption of 36.4 g/d in Cameroon and Arth's threshold of 150 μg/d, the predicted number of NTD cases averted would double at the wheat flour fortification level of 4.12 μg/g (calculated as 150 μg/d divided by 36.4 g/d). According to Wald's model (8), the predicted number of NTD cases averted increased as the fortification level increased but did not reach a plateau. Overall, in terms of NTD cases averted, the predictions by Arth's model were substantially higher than those from other models at all fortification levels, whereas the predictions from Wald's model were the lowest.

Two indirect models, Wald's (8) and Crider's 2019 (11) models, allow predictions of the change in plasma folate concentration due to fortification. We were able to assess the outputs of these 2 models by comparing the predicted plasma folate concentrations with the observed values obtained in 2 major cities in Cameroon 1 y after initiation of the wheat flour fortification program (31). Wald's model (8) predicted a slightly higher plasma folate concentration than Crider's 2019 model (11) at the simulated fortification levels presented (Figure 2B). Both models predicted plasma folate concentrations below the pF-IT of 25.5 nmol/L (12) for Cameroon, which was substantially lower than the mean concentration (46.9 nmol/L) observed in 2 major cities after folic acid fortification.

## Comparison of Existing Models to Predict NTD Reduction Due to Folic Acid Fortification

Our third objective was to compare these models based on the plausibility of their predictions. Formal validation of the models would require collection of primary data on individual folate intake and/or status and NTD birth prevalence prior to and after fortification programs. Thus, we are not able to assess the true validity of the models due to limited data availability and the large sample size that would be required for the primary research. Instead, we elected to judge the likely accuracy of results based on 3 principles. First, the baseline data should be well conceived and of good quality. Because folic acid fortification can prevent both NTD-affected live births and stillbirths, the baseline NTD prevalence should be all NTD cases, regardless of whether they were stillborn or live births (1). Second, current evidence indicates that NTDs cannot be entirely prevented by folic acid fortification, and the lowest achievable NTD prevalence following introduction of folic acid fortification is ∼5.0 per 10,000 live births, which is the NTD birth prevalence observed in several countries considered to have well-functioning fortification programs (7). Third, we would expect the predicted number of NTDs averted to increase as fortification levels increase and then ultimately reach a plateau (26, 33) when all WRA reach the respective clinical biomarker thresholds for sufficiency [i.e., the 906-nmol/L RBC folate threshold (18), or the estimated pF-IT (12)]. It is possible that the simulated fortification levels might not be high enough to reach a plateau in the number of NTDs prevented; however, a constant percentage of NTDs averted with increased fortification levels or an abrupt fall in NTD prevalence at a specific intake level were deemed to be unlikely.

With the existing data, we are able to evaluate the 2 indirect approach models, Wald's (8) and Crider's 2019 (11) models, by comparing the model results with observed plasma folate concentration collected after 1 y of the national wheat flour fortification program in 2 major cities in Cameroon (31). We then evaluated whether the predicted plasma folate concentrations from Wald's (8) and Crider's 2019 (11) models surpass the pf-IT (12), which would indicate the likelihood of reducing NTD cases. We would expect the plasma folate concentration to increase as fortification levels rise.

### Models that predict NTDs averted

In this section, 4 models that predict NTDs averted are evaluated based on the 3 principles stated above. The original LiST model (6) only estimates the effect of folic acid fortification on neonatal deaths. Moreover, the benefits of folic acid fortification were applied to the proportion of the population that consumed any fortified food (reach), independent of the amount of fortified food consumed. Hence, the predicted percentage of NTDs averted was constant regardless of fortification level, which is contrary to biological plausibility (9). The modified LiST model represents an improvement over the original LiST model because it includes NTD deaths during and beyond the neonatal period, stillbirths, and (for countries with antenatal screening programs) NTD-related eTOPFA. Furthermore, the modified LiST model is advantageous in that it can apply different measures that approximate the percentage of the population affected by the folic acid–fortification program (reach vs. % reduction in inadequate intake) to predict the number of NTDs averted. The modified LiST model estimate #2 predicted that the percentage of NTDs averted would increase as the fortification level increases and then reach a plateau at higher levels of fortification. This latter scenario is more consistent with what would be expected biologically. However, even though the number of NTDs averted reaches a plateau according to the modified LiST model estimate #2, the estimates from this model do not attain the lowest achievable NTD prevalence, as the model imposes a maximum of 62% effectiveness, regardless of the baseline NTD prevalence.

Arth's model (7) has both strengths and limitations. After 20 y of fortification and high wheat flour consumption in the United States, the NTD prevalence is still 5.0 per 10,000 live births, suggesting that NTDs cannot be fully prevented by folic acid fortification alone (34). Arth's model clearly captures this fact and further indicates that the percentage of NTDs that are folic acid preventable depends on the prevalence of NTDs prior to fortification. However, according to Arth's model, the predicted number of NTDs averted abruptly doubles when additional folic acid intake surpasses the 150-μg/d threshold. First, this 150 μg/d was based on the expert opinion of the Teratology Society instead of published research. The suggested daily consumption threshold of 150 μg folic acid/d (23) for NTD prevention is considerably less than the recommendations of the CDC (35) and the Institute of Medicine (36), which propose that women should consume 400 μg folic acid per day, in addition to folate intake from food. Furthermore, the estimated percentage of NTDs averted is unlikely to have such a sudden and steep response once the population mean additional folic acid intake crosses a specific intake threshold. In addition, the original Arth model used data from the FAO Food Balance Sheets to estimate average wheat flour consumption per capita, which is then used to calculate the average additional folic acid intake (37). However, the wheat flour consumption estimated using the Food Balance Sheets only represents the national average wheat flour availability and not the usual wheat flour consumption by a specific population subgroup (e.g., WRA). In our application of Arth's model to the data from Cameroon, we used 24-h dietary recall data to calculate usual wheat flour consumption among women to reduce this error imposed by applying national per capita average food availability to represent dietary intake among WRA (37).

The Wald model (8) predicts a gradual, continuous change in percentage of NTDs averted with increasing folic acid intake. According to this model, the maximum percentage of NTDs preventable by folic acid fortification could be close to 100%, which is unlikely. Second, extrapolation of the model yields implausible results: to achieve a reduction of >90% of NTDs, the additional amount of folic acid consumed via wheat flour implied by this equation would be 13 mg/d. This amount is 13 times the Tolerable Upper Intake Level for folic acid and unlikely to be achieved even with use of high-dose supplements. Wald et al. also assessed their model by comparing the estimates from their model with results from other intervention trials and observational studies; their results are consistent with results from 3 direct observations: *1*) the randomized double-blind trials carried out in 33 centers in 7 different countries by the Medical Research Council vitamin study (38), *2*) a meta-analysis of case-control studies (39), and *3*) the US folic acid fortification program (40). However, Wald's model underestimated the effect of folic acid on NTDs using data from a center-based folic acid supplementation trial in the United Kingdom (41) and the Community Intervention Project conducted in northern (Hebei Province) and southern (Zhejiang and Jiangsu Provinces) regions of China from 1993 through 1995 (26). In our review, we also found that Wald's model underestimated the impact of the folic acid–fortification program on plasma folate concentration in urban Cameroon; thus, it would likewise underestimate the percentage of NTDs averted.

Arth's (7) and Wald's (8) models use estimates of the population mean additional folic acid intake, which, in this analysis, was calculated using mean usual wheat flour intake from individual 24-h recalls. As individual wheat flour consumption in Cameroon was highly right-skewed (13), using the population average of wheat flour consumption could overestimate the amount of daily folic acid intake for the majority of the population. However, as we stated previously, this estimate may still be better than alternatives, such as reliance on FAO Food Balance Sheets or industry data on wheat flour production to estimate individual intake.

### Indirect models that predict plasma folate concentration

The 2 indirect models, Wald's (8) and Crider's 2019 models (11), both underpredicted plasma folate concentration change from fortified wheat flour. Even though it is possible that wheat flour intake, and thus folic acid intake, was underestimated in the prefortification dietary survey or that wheat flour intake was estimated correctly and subsequently increased, it does not seem likely that the large difference between the model's prediction and the observed plasma folate concentration in Cameroon can be fully explained by increased flour consumption. To achieve the observed increase in plasma folate concentration in 2 major cities, according to Wald's model (8), women would need to have an additional average folic acid consumption of 11,888 μg/d, which is an equivalent of 2.4 kg/d of wheat flour consumption; according to Crider's 2019 model (11), women need to consume 1051 μg/d additional folic acid, equivalent to 210 g/d additional wheat flour consumption, which is more reasonable, but still high. Hence, it is likely that both Wald's and Crider's 2019 models underestimated the impact of folic acid fortification programs on plasma folate concentration. The implications of this discrepancy may be important for programs: in this case study, the predicted plasma folate concentration was far below the pf-IT (12), implying that additional population-based intervention would be needed for NTD prevention, whereas the observed plasma folate concentration was substantially greater than the pf-IT, indicating that no other interventions are needed.

## Discussion

NTDs are responsible for a considerable number of early childhood deaths and disabilities globally, especially in LMICs, so greater effort is needed to expand relevant public health programs. Predictive models of the potential effect of additional folic acid intake on NTD risk can assist program planning and advocacy efforts, but discrepancies in the outputs of existing models may cause confusion and, at worst, delay programmatic action. Our literature review identified 7 models that were developed to estimate the impact of folic acid–fortification programs on NTD risk, but the models yielded widely varying results when applied to data from Cameroon, and some of the results were biologically implausible. In practice, data availability may determine which model is applied.

**FIGURE 3:**
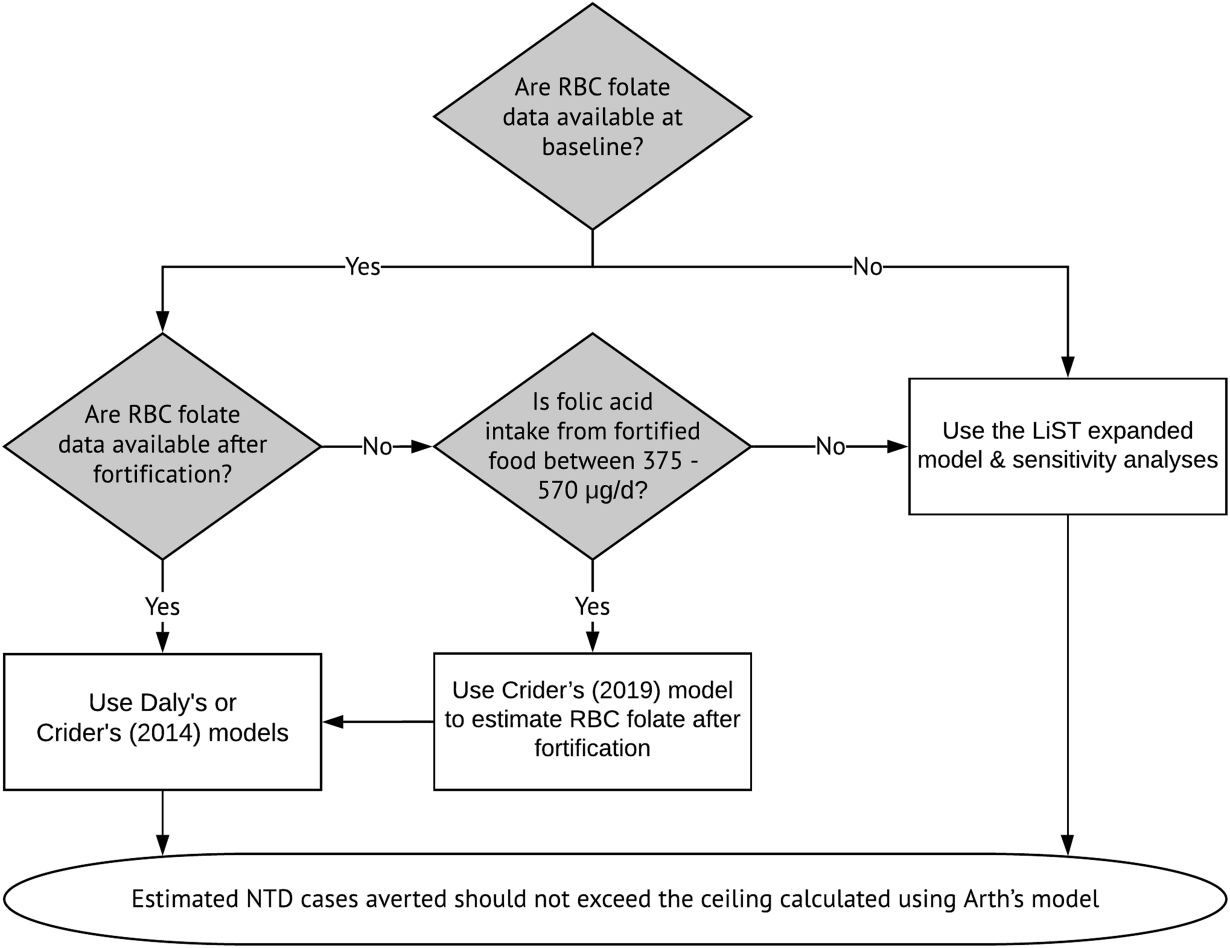
Decision-making tree in selecting the NTD-predictive model based on data availability. We only include NTD-predictive models used in the decision-making tree in selecting the optimal NTD-predictive models. The original LiST and Wald models that predict NTDs averted and Crider's (2019) model that predicts plasma folate concentration change due to fortification are not included in the decision-making tree. LiST, Lives Saved Tool; NTD, neural tube defect.

Based on the criteria of biological plausibility and consistency with empirical data, we developed a decision-making tree (**Figure 3**) for predicting NTDs averted. Users should first calculate the ceiling of NTD cases averted based on Arth's model, which is equal to the maximum proportion of NTDs that can be prevented by folic acid fortification multiplied by the number of live births [i.e., (prevalence of NTDs − 5.0 per 10,000 live births) × the number of live births]. The final number of predicted NTD cases averted by fortification should not exceed this ceiling. If the model prediction is less than the ceiling, the model result is the final predicted NTD cases averted. If the model prediction exceeds the ceiling, the final number of NTD cases averted would be assumed to be the same as the ceiling.

When data on RBC folate concentrations at baseline and after fortification are available, our review suggests that the best practice is to estimate the NTD cases averted by using Daly (9) and Crider's 2014 (10) models and then compare the results with the ceiling of the NTD cases averted. A limitation of our analysis is that RBC folate concentrations were not available for women in Cameroon, so Daly's (9) and Crider's 2014 (10) models were not included in the comparison of model results. Although we could not compare the results directly, these models are based on biologically plausible assumptions and supported by empirical data, and the predictions of the 2 models are consistent according to Crider's 2014 paper (10). Specifically, there is a clear relation between RBC folate concentration (a preferred indicator of longer-term folate status) and NTD risk (9, 10).

When the RBC folate concentration at baseline is available but the RBC concentration after fortification is not available, we first recommend users estimate additional folic acid intake by using individual dietary intake data (rather than national estimates such as those from Food Balance Sheets). If the amount of additional folic acid intake falls within the range of 375 and 570 μg/d (11), users can first estimate the RBC folate concentration after fortification by applying Crider's 2019 model (11) with use of information on *1*) additional folic acid intake and *2*) the baseline RBC folate concentration, and then use the baseline and the predicted RBC folate concentrations to estimate NTD risk. The predicted RBC folate concentration is based on solid empirical information, as long as the amount of additional folic acid intake falls within the range of 375 and 570 μg/d (11). This dosage may be more consistent with supplementation programs than with fortification programs, but, conceivably, the models could be applied for folic acid intakes outside this range, albeit with greater uncertainty.

Unfortunately, representative data on RBC folate concentrations are available for a relatively small proportion of LMICs (17), although efforts are underway to rectify this scarcity of information (42, 43). When the baseline RBC concentration is not available, or when the baseline RBC concentration is available but the additional folic acid intake is outside the range of 375 and 570 μg/d, the modified LiST model estimate #2 may be the most appropriate model. This is because the modified LiST estimate #2 appears to best capture the predicted gradual increase and then plateau in NTD cases averted as fortification levels are increased or the proportion of the population reached increases. However, the modified LiST estimate #2 (6) ignores the fact that the percentage reduction in NTDs depends on the baseline NTD prevalence in the population (7). Therefore, the modified LiST model has important limitations and should be interpreted with caution. To address these limitations, users should carry out sensitivity analyses to describe a range of possible outcomes. For example, when the baseline RBC concentration is available, but the additional folic acid intake is outside of the range of 375–570 μg/d, users could still use Crider's 2019 model (11) to predict the RBC folate concentration after fortification, and then apply Daly (9) and Crider's 2014 (10) models to predict NTD cases averted.

We did not include models estimating the relation between folic acid intake and plasma folate concentration and the subsequent effect on NTD risk [i.e., Wald's model (8) or part of Crider's 2019 model (11) that predicts the plasma folate concentration due to fortification] in the decision-making tree, because the relation between plasma folate concentration and NTD risk is not well defined (12) compared with models that use RBC folate. In addition, the relation between folic acid intake and plasma folate was not supported by the results of our case study, as both Wald's (8) and Crider's 2019 (11) models underestimated the plasma folate changes due to folic acid fortification in Cameroon. Thus, there remains some uncertainty about the quantitative effects of folic acid intake on NTD risk via the folic acid intake–plasma folate–NTD pathway.

Estimations of the additional folic acid intake provided by fortification ideally should be based on individual intake of the fortification vehicle and any other sources of folic acid, including supplements, using quantitative dietary intake assessments. While some models rely on national estimates based on Food Balance Sheets or industry data, these numbers are less accurate than individual intake data and do not capture the distribution of intakes among different subgroups of the population. Thus, when data are available from dietary surveys, this information is likely to improve the quality of estimates of the effect of fortification or other programs to deliver folic acid on reductions in NTDs.

A limitation of the present analysis is that we did not present estimates of error or uncertainty around the modeled point estimates. While we acknowledge that attempts have been made to estimate UIs around some of the input parameters [i.e., the estimates of neonatal deaths from congenital anomalies due to NTDs by Blencowe and colleagues (4)], many of the model parameters do not. For example, estimates of population average folic acid consumption that rely on Food Balance Sheets data do not have an associated UI, nor does the minimum threshold for folic acid–preventable NTD prevalence. Therefore, we urge caution in interpreting the precise point estimates derived through each of these models. For models for which all parameters have an associated estimate of uncertainty, appropriate statistical methods are needed to ensure that the final estimates reflect the uncertainty in each of the parameters.

In conclusion, models of the relation between folic acid intake and NTD reduction could help drive policy decisions regarding folic acid fortification and guide the design of new intervention programs. When appropriate data are available, the best practice to estimate folic acid–preventable NTDs is to apply Daly (9) and Crider's 2014 models (10) by using the RBC folate concentration at the baseline and after fortification. When the baseline RBC folate concentration is available but the RBC folate concentration after fortification is not available, users should first estimate the effect of increased folic acid intake on RBC folate concentration and then use the predicted RBC folate concentration to estimate NTD risk (although additional information is needed to predict RBC folate concentration when the mean folic acid intake is <375 μg/d) (11). When data on RBC folate concentration are not available, policymakers and advocates may consider applying the modified LiST (6) estimate #2 but should be aware that the results from the modified LiST Estimate #2 may not agree with the reality and that estimates based on RBC folate provide more certainty, although greater investment is required to generate the information. Further research is needed to validate models describing the relation between folic acid intake, markers of folate status, and NTD risk. In the meantime, sensitivity analyses (i.e., the application of several models to generate a range of predictions) may be useful to describe a range of possible outcomes. Collection of appropriate biomarkers, improved estimation of individual intake of folic acid, and implementing or strengthening surveillance systems for birth defects and neonatal mortality in LMICs could improve model inputs and model utility for decision making.

## Supplementary Material

nmab083_Supplemental_File

## Notes

This work was supported, in whole or in part, by the Bill & Melinda Gates Foundation [OPP1170661]. Under the grant conditions of the Foundation, a Creative Commons Attribution 4.0 Generic License has already been assigned to the Author Accepted Manuscript version that might arise from this submission. This analysis was supported in part by the National Cancer Institute. Collection and analysis of the dietary data were supported by the Michael and Susan Dell Foundation, Sight and Life, UNICEF, and the Bill & Melinda Gates Foundation.

Author disclosures: The authors report no conflicts of interest.

Supplemental Figures 1–3 and Supplemental Methods 1 are available from the “Supplementary data” link in the online posting of the article and from the same link in the online table of contents at https://academic.oup.com/advances/.

Abbreviations used: CNMS, Cameroon National Micronutrient Survey; eTOPFA, elective termination of pregnancy for fetal anomalies; LiST, Lives Saved Tool; LMIC, low- and middle- income country; NTD, neural tube defect; pf-IT, plasma folate insufficiency threshold; UI, uncertainty interval; WRA, Women of Reproductive Age.

## References

[1] Blencowe H , Kancherla V, Moorthie S, Darlison MW, Modell B. Estimates of global and regional prevalence of neural tube defects for 2015: a systematic analysis. Ann N Y Acad Sci. 2018;1414:31–46. 29363759 10.1111/nyas.13548

[2] Liu S , West R, Randell E, Longerich L, O'Connor KS, Scott H, Crowley M, Lam A, Prabhakaran V, McCourt C. A comprehensive evaluation of food fortification with folic acid for the primary prevention of neural tube defects. BMC Pregnancy Childbirth. 2004;4:20. 15450123 10.1186/1471-2393-4-20 PMC524178

[3] Atta CAM , Fiest KM, Frolkis AD, Jette N, Pringsheim T, St Germaine-Smith C, Rajapakse T, Kaplan GG, Metcalfe A. Global birth prevalence of spina bifida by folic acid fortification status: a systematic review and meta-analysis. Am J Public Health. 2016;106:e24–34. 26562127 10.2105/AJPH.2015.302902 PMC4695937

[4] Blencowe H , Cousens S, Modell B, Lawn J. Folic acid to reduce neonatal mortality from neural tube disorders. Int J Epidemiol. 2010;39(Suppl 1):i110–21. 20348114 10.1093/ije/dyq028 PMC2845867

[5] Garrett GS , Bailey LB. A public health approach for preventing neural tube defects: folic acid fortification and beyond. Ann N Y Acad Sci. 2018;341:155–12. 10.1111/nyas.13579 29450891

[6] Walker N , Tam Y, Friberg IK. Overview of the Lives Saved Tool (LiST). BMC Public Health. 2013;13:S1. 10.1186/1471-2458-13-S3-S1 PMC3847271 24564438

[7] Arth A , Kancherla V, Pachón H, Zimmerman S, Johnson Q, Oakley GP Jr. A 2015 global update on folic acid-preventable spina bifida and anencephaly. Birth Defect Res A. 2016;106:520–9. 10.1002/bdra.23529 27418029

[8] Wald NJ , Law MR, Morris JK, Wald DS. Quantifying the effect of folic acid. Lancet North Am Ed. 2001;358:2069–73. 10.1016/s0140-6736(01)07104-5 11755633

[9] Daly LE , Kirke PN, Molloy A, Weir DG, Scott JM. Folate levels and neural tube defects: implications for prevention. JAMA. 1995;274:1698. 7474275 10.1001/jama.1995.03530210052030

[10] Crider KS , Devine O, Hao L, Dowling NF, Li S, Molloy AM, Li Z, Zhu J, Berry RJ. Population red blood cell folate concentrations for prevention of neural tube defects: Bayesian model. BMJ. 2014;349:g4554–4. 25073783 10.1136/bmj.g4554 PMC4115151

[11] Crider K , Devine O, Qi Y, Yeung L, Sekkarie A, Zaganjor I, Wong E, Rose C, Berry R. Systematic review and Bayesian meta-analysis of the dose-response relationship between folic acid intake and changes in blood folate concentrations. Nutrients. 2019;11:71–14. 10.3390/nu11010071 PMC6356991 30609688

[12] Chen M-Y , Rose CE, Qi YP, Williams JL, Yeung LF, Berry RJ, Hao L, Cannon MJ, Crider KS. Defining the plasma folate concentration associated with the red blood cell folate concentration threshold for optimal neural tube defects prevention: a population-based, randomized trial of folic acid supplementation. Am J Clin Nutr. 2019;145:1636s–10. 10.1093/ajcn/nqz027 PMC7099800 31005964

[13] Engle-Stone R , Ndjebayi AO, Nankap M, Brown KH. Consumption of potentially fortifiable foods by women and young children varies by ecological zone and socio-economic status in Cameroon. J Nutr. 2012;142:555–65. 22323765 10.3945/jn.111.148783

[14] Njamnshi AK , Djientcheu V de P, Lekoubou A, Guemse M, Obama MT, Mbu R, Takongmo S, Kago I. Neural tube defects are rare among black Americans but not in sub-Saharan black Africans: the case of Yaounde–Cameroon. J Neurol Sci. 2008;270:13–7. 18295800 10.1016/j.jns.2008.01.010

[15] Shahab-Ferdows S , Engle-Stone R, Hampel D, Ndjebayi AO, Nankap M, Brown KH, Allen LH. Regional, socioeconomic, and dietary risk factors for vitamin B-12 deficiency differ from those for folate deficiency in Cameroonian women and children. J Nutr. 2015:145(11):2587–95. 26446486 10.3945/jn.115.210195

[16] Martinez H , Weakland AP, Bailey LB, Botto LD, De-Regil LM, Brown KH. Improving maternal folate status to prevent infant neural tube defects: working group conclusions and a framework for action. Ann NY Acad Sci. 2018;1414:5–19. 29532514 10.1111/nyas.13593

[17] Bailey LB , Stover PJ, McNulty H, Fenech MF, Gregory JF, Mills JL, Pfeiffer CM, Fazili Z, Zhang M, Ueland PM et al. Biomarkers of Nutrition for Development—folate review. J Nutr. 2015;145:1636S–80S. 26451605 10.3945/jn.114.206599 PMC4478945

[18] World Health Organization . Guideline: optimal serum and red blood cell folate concentrations in women of reproductive age for prevention of neural tube defects. Geneva (Switzerland): World Health Organization; 2015. 25996016

[19] Werler MM , Shapiro S, Mitchell AA. Periconceptional folic acid exposure and risk of occurrent neural tube defects. JAMA. 1993;269:1257–61. 8437302

[20] Kancherla V , Black RE. Historical perspective on folic acid and challenges in estimating global prevalence of neural tube defects. Ann N Y Acad Sci. 2018;1414:20–30. 29532513 10.1111/nyas.13601

[21] Marchetta C , Devine O, Crider K, Tsang B, Cordero A, Qi Y, Guo J, Berry R, Rosenthal J, Mulinare J et al. Assessing the association between natural food folate intake and blood folate concentrations: a systematic review and Bayesian meta-analysis of trials and observational studies. Nutrients. 2015;7:2663–86. 25867949 10.3390/nu7042663 PMC4425166

[22] Mosley BS , Cleves MA, Siega-Riz AM, Shaw GM, Canfield MA, Waller DK, Werler MM, Hobbs CA; National Birth Defects Prevention Study. Neural tube defects and maternal folate intake among pregnancies conceived after folic acid fortification in the United States. Am J Epidemiol. 2009;169:9–17. 18953063 10.1093/aje/kwn331 PMC3139973

[23] Smith MA , Lau C. A resolution on folic acid fortification. Birth Defect Res A. 2015;103:1–2. 10.1002/bdra.23339 25516484

[24] Food Fortification Initiative . Global progress [Internet]. [cited 2016 Nov 22]. Available from: https://www.ffinetwork.org/globalprogress.

[25] Kirke PN , Molloy AM, Daly LE, Burke H, Weir DG, Scott JM. Maternal plasma folate and vitamin B12 are independent risk factors for neural tube defects. Q J Med. 1993;86:703–8. 8265769

[26] Berry RJ , Li Z, Erickson JD, Li S, Moore CA, Wang H, Mulinare J, Zhao P, Wong LY, Gindler J et al. Prevention of neural-tube defects with folic acid in China. China-U.S. Collaborative Project for Neural Tube Defect Prevention. N Engl J Med. 1999;341:1485–90. 10559448 10.1056/NEJM199911113412001

[27] Hao L , Yang QH, Li Z, Bailey LB. Folate status and homocysteine response to folic acid doses and withdrawal among young Chinese women in a large-scale randomized double-blind trial. Am J Clin Nutr. 2008;88:448–57. 18689382 10.1093/ajcn/88.2.448

[28] Gibson RS , Ferguson EL. An interactive 24-hour recall for assessing the adequacy of iron and zinc intakes in developing countries. HarvestPlus Technical Monograph 8. Washington, DC and Cali: International Food Policy Research Institute (IFPRI) and International Center for Tropical Agriculture (CIAT); 2008.

[29] United Nations Inter-agency Group for Child Mortality Estimation . Child mortality estimates. Available from: childmortality.org. 10.1371/journal.pone.0101112 PMC4094389 25013954

[30] United Nations Department of Economic and Social Affairs, Population Division . UNpop [Internet]. [cited 7 September, 2020]. Available from: https://esa.un.org/unpd/wpp/Download/Standard/Population/.

[31] Engle-Stone R , Nankap M, Ndjebayi AO, Allen LH, Shahab-Ferdows S, Hampel D, Killilea DW, Gimou M-M, Houghton LA, Friedman A et al. Iron, zinc, folate, and vitamin B-12 status increased among women and children in Yaoundé and Douala, Cameroon, 1 year after introducing fortified wheat flour. J Nutr. 2017;147(7):1426–36. 28592513 10.3945/jn.116.245076 PMC5483962

[32] World Health Organization . Recommendations on wheat and maize flour fortification. Geneva (Switzerland): World Health Organization; 2011.

[33] Seller MJ , Nevin NC. Periconceptional vitamin supplementation and the prevention of neural tube defects in south-east England and Northern Ireland. J Med Genet. 1984;21:325–30. 6502647 10.1136/jmg.21.5.325 PMC1049312

[34] US Preventive Services Task Force; Bibbins-Domingo K, Grossman DC, Curry SJ, Davidson KW, Epling JW Jr, García FAR, Kemper AR, Krist AH, Kurth AE et al. Folic acid supplementation for the prevention of neural tube defects. JAMA. 2017;317:183–7. 28097362 10.1001/jama.2016.19438

[35] Centers for Disease Control . Recommendations for the use of folic acid to reduce the number of cases of spina bifida and other neural tube defects. MMWR: Morbidity and Mortality Weekly Report. Centers for Disease Control; 1992. p. 1–7. 1522835

[36] Institute of Medicine . Folate. Dietary Reference Intakes for thiamin, riboflavin, niacin, vitamin B6, folate, vitamin B12, pantothenic acid, biotin, and choline. Washington (DC): National Academies Press; 1998. 23193625

[37] Food and Agriculture Organization of the United Nations . FAOSTAT. Food Balance Sheet. [Internet]. Available from: http://data.fao.org/ref/48dc9161-53e2-4883-93c0-8f099e5e67ab.html?version=1.0.

[38] MRC Vitamin Study Research Group . Prevention of neural tube defects: results of the Medical Research Council Vitamin Study. Lancet North Am Ed. 1991;338:131–7. 1677062

[39] Wald NJ . Folic acid and the prevention of neural tube defects. Ann NY Acad Sci. 1993;678:112–29. 8494254 10.1111/j.1749-6632.1993.tb26114.x

[40] Rader JI , Weaver CM, Angyal G. Advances in the analysis of folates in foods. Food Testing & Analysis. 1999;5(2):14–32.

[41] Smithells RW , Seller MJ, Harris R, Fielding DW, Schorah CJ, Nevin NC, Sheppard S, Read AP, Walker S, Wild J. Further experience of vitamin supplementation for prevention of neural tube defect recurrences. Lancet North Am Ed. 1983;321:1027–31. 10.1016/s0140-6736(83)92654-5 6133069

[42] Botto LD , Mastroiacovo P. Triple surveillance: a proposal for an integrated strategy to support and accelerate birth defect prevention. Ann NY Acad Sci. 2018;1414:126–36. 29532515 10.1111/nyas.13600 PMC5873412

[43] Martinez H , Poulin J, Weakland A, Bailey L, Mehta R, Afidra R, Brown K. Developing a global strategy for the control of folate deficiency and folic acid responsive neural tube defects in low- and middle-income countries. Curr Dev Nutr. 2019;3:nzz034.

