# Supplementary material for: Review of Existing Models to Predict Reductions in Neural Tube Defects Due to Folic Acid Fortification and Model Results Using Data from Cameroon
Source: Adv Nutr. 2021 Jul 19;12(6):2401–14. doi: 10.1093/advances/nmab083 (PMC8634386; doi:10.1093/advances/nmab083)
Supplement: nmab083_Supplemental_File [file nmab083_supplemental_file.docx]

**Supplementary figure 1:**

Pathway between folic acid fortification and NTD-affected neonatal deaths averted using the LiST original model lower bound (Estimate #1) (A) and higher bound (Estimate #2) (B)^1^


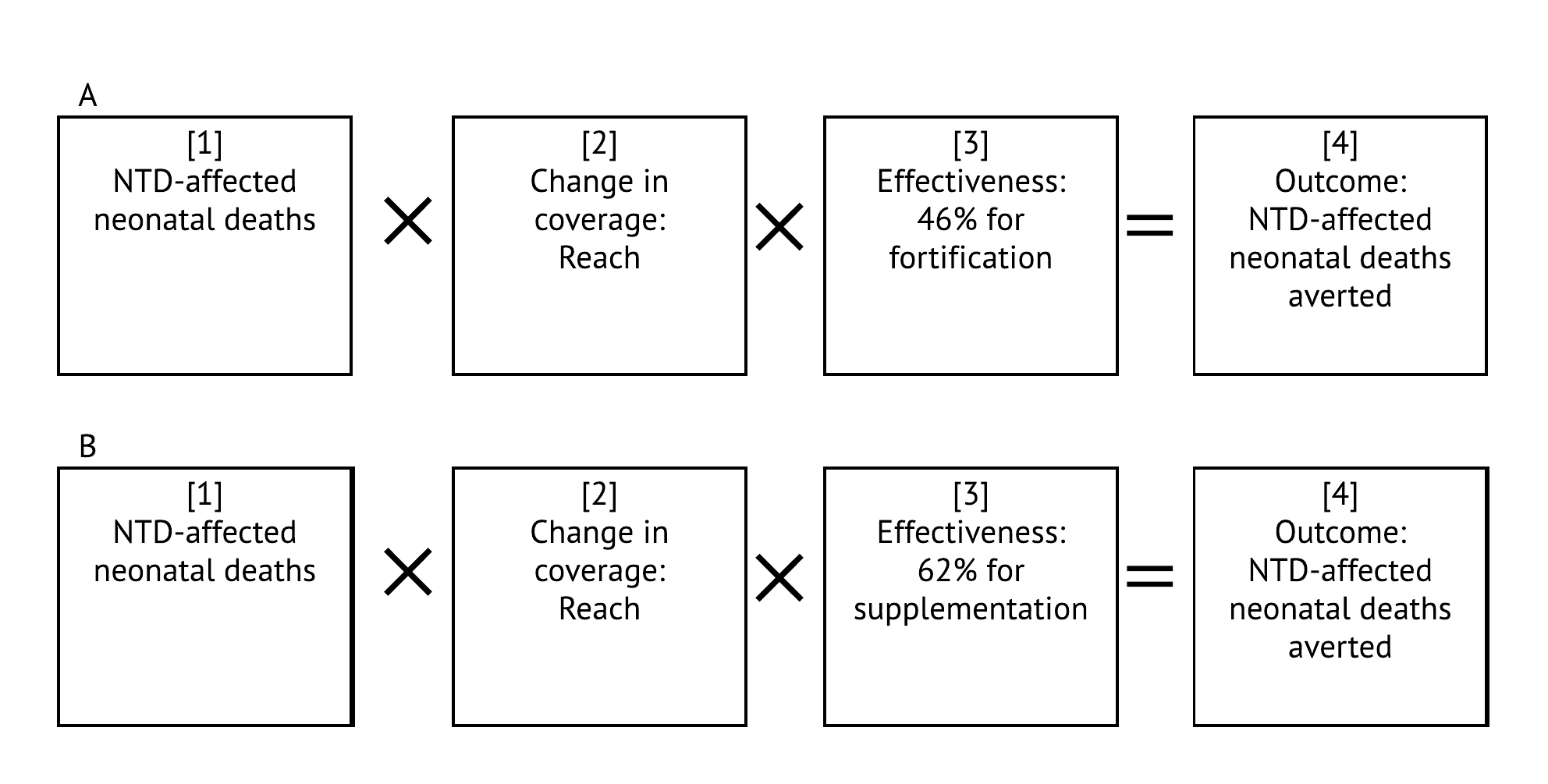


^1^LiST, Lives Saved Tool; NTD, Neural Tube Defect.

**Supplementary figure 2:**

Pathway between folic acid fortification and NTD cases averted using the LiST modified model Estimate #1 (reach x fortification effectiveness) (A) and Estimate #2 (reduction in inadequate intake x supplementation effectiveness) (B) ^1^


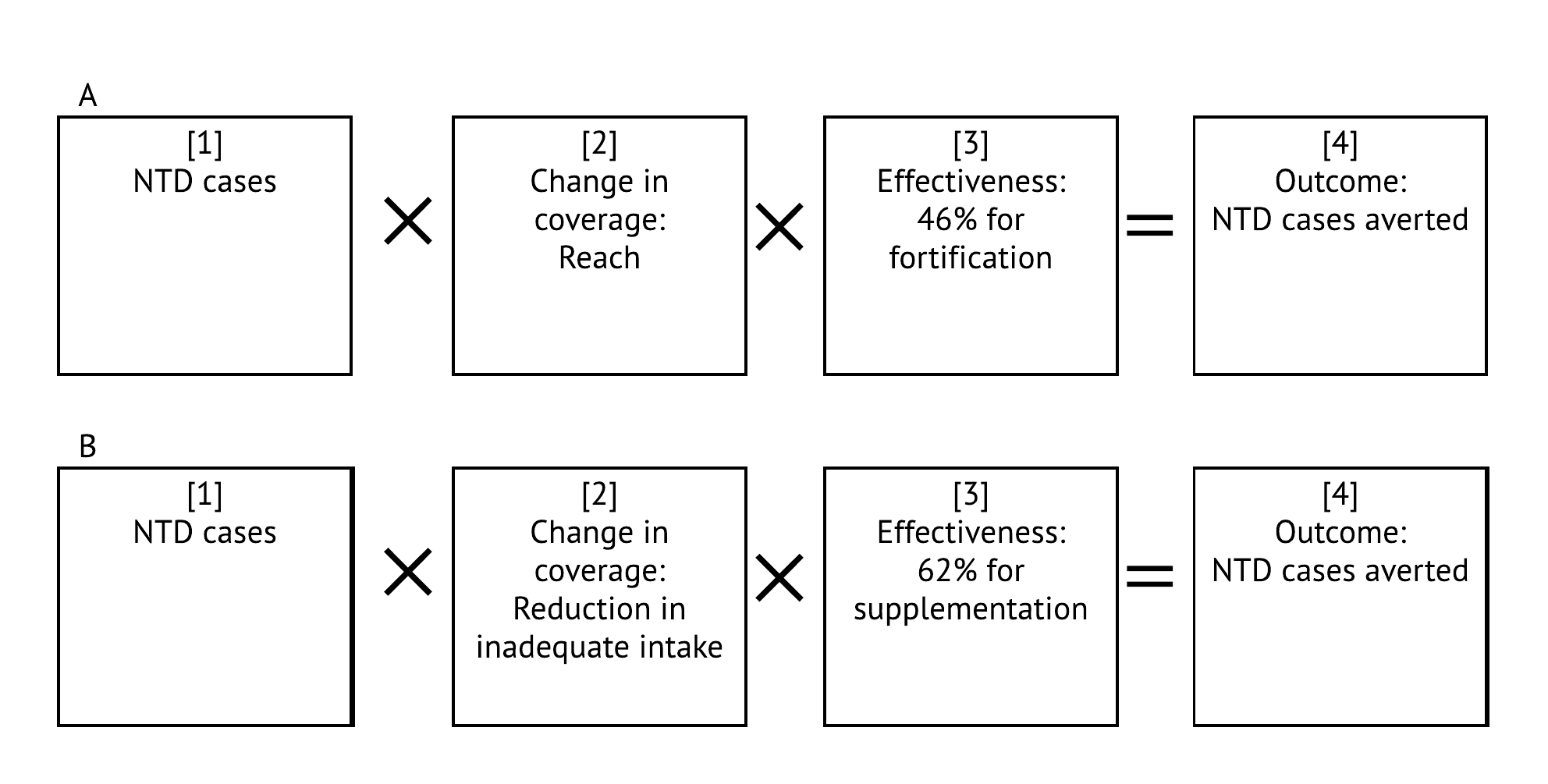


^1^LiST, Lives Saved Tool; NTD, Neural Tube Defect.

**Supplementary figure 3:**

Pathway between folic acid fortification and NTDs averted through the Arth’s model^1^

^
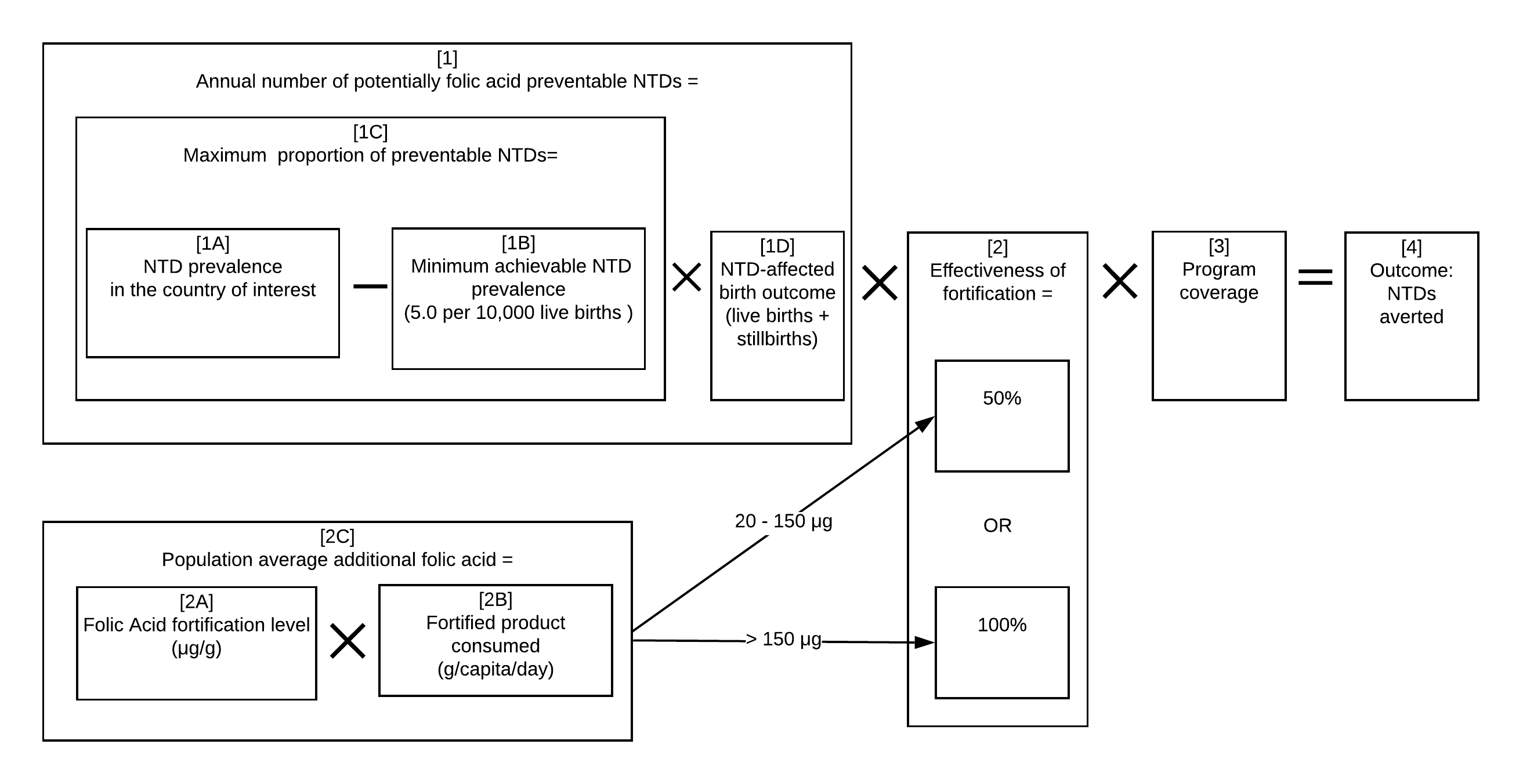
^

^1^NTD, Neural Tube Defect.

**Supplementary Method 1**

**Application of models to Cameroon National Micronutrient Survey**

A detailed description of the methods for applying each of the models to the available data from Cameroon is presented below.

1. **Original LiST model.** The original LiST model (1) estimates NTD-affected neonatal deaths averted following folic acid fortification by multiplying NTD-related neonatal deaths at baseline by 1) the coverage of the intervention and 2) a value representing the assumed effectiveness of folic acid fortification for reducing NTD-related deaths. The analytical method of the original LiST model is also demonstrated in **Supplementary Figure 1**. Given that there were no folic acid-fortified staple foods in Cameroon prior to the initiation of the mandatory wheat flour fortification program and assuming that unfortified wheat flour could be completely replaced by the fortified product, we defined the change of program coverage as the program’s reach, which is the percentage of women consuming any amount of folic acid fortified wheat flour on the previous day. Two different estimates of effectiveness (46% and 62%) were applied as the lower and higher bounds of the original LiST model, as explained above. Hence, the lower bound of NTDs averted due to fortification (Estimate #1) is [248 NTD-affected neonatal deaths] x [45.9% reach] x [46% effectiveness] = 52 deaths averted. Similarly, the upper bound of NTD deaths averted (Estimate #2) is 248 x 45.9% x 62% = 71 deaths averted.
2. **Modified LiST model.** Following a similar approach as was used for the original LiST model (1), we developed a modified LiST model to estimate NTD cases averted from folic acid fortification by multiplying NTD cases (instead of NTD-affected neonatal deaths) at baseline by 1) the change in coverage of folic acid interventions and 2) effectiveness of providing additional folic acid. The analytical method of the modified LiST model is also demonstrated in **Supplementary Figure 2**.

In contrast to the original LiST model, we used two values for change in coverage for the modified LiST model. Because reach merely evaluates whether individuals consumed fortified flour recently, but does not evaluate whether the consumed amount of flour provides a sufficient dose of folic acid for NTD prevention, we defined a second metric for change in coverage: percent reduction in inadequate intake, which is the percent of the total population who have shifted from inadequate to adequate intakes following introduction of fortified wheat flour (5 µg folic acid per g of wheat flour), with inadequate intake defined as intake below the estimated average requirement. For the first set of estimates (Estimate #1), we multiplied reach by the LiST-specified effectiveness value for fortification programs (46% effectiveness). For the second set of estimates (Estimate #2), we applied percent reduction in inadequate intake and the effectiveness value based on supplementation trials (62% effectiveness). Thus, the two sets of estimated NTD cases averted are 1) [2,334 NTD cases] x [45.9% reach] x [46% effectiveness for fortification] = 493 cases averted; and 2) [2,334 NTD cases] x [50.1% reduction in inadequate intake] x [62% effectiveness for supplementation] = 724 cases averted.

1. **Modified Arth’s model**. Arth’s model (2) estimates NTD cases averted due to a fortification program by multiplying the annual number of NTDs potentially preventable by folic acid by 1) the effectiveness of the fortification program based on the estimated amount of additional folic acid intake and 2) the program coverage. The analytical method of Arth’s model is presented in **Supplementary Figure 3**. According to Arth’s model, the number of NTD cases potentially preventable by folic acid is calculated by multiplying the maximum proportion of preventable NTDs by the annual number of live births. The original Arth model (2) only includes the number of live births, so for the comparison with data from Cameroon (described below) we also included stillbirths in the modified model, as described above for the modified LiST model. The maximum proportion of NTDs that can be prevented by folic acid fortification is calculated as the difference in NTD prevalence between the country of interest and the minimum achievable NTD prevalence of 5.0/10,000 live births. The NTD prevalence in Cameroon is 29.5/10,000 live births, as presented above. Therefore, the maximum proportion of NTD that can be prevented by folic acid fortification is 29.5 – 5.0 = 24.5 NTDs per 10,000 live births. Then, the number of folic acid preventable NTD cases is calculated by multiplying maximum proportion of preventable NTD by the number of live births and stillbirths, which is [24.5 NTDs per 10,000 live births] X [790,346 live births and stillbirths] = 1,936 cases.

The effectiveness of the folic acid fortification program (that is, the proportion of NTD cases prevented) is estimated based on the population average additional folic acid consumption. To calculate the *population average additional folic acid consumption*, we applied the National Cancer Institute (NCI) method (3) to estimate the population’s usual fortified wheat flour intake in Cameroon (4). Then, we calculated the amount of additional folic acid consumed through fortification by multiplying the usual mean wheat flour intake (in g/d) by the fortification level (5 µg folic acid per g of wheat flour), with the assumption that unfortified wheat flour was completely replaced by the fortified product. Based on the measured amount of wheat flour intake and the target level of folic acid fortification, the average additional folic acid consumption in Cameroon is [36.4 g usual wheat flour consumption] x [5 µg folic acid per g of wheat flour] = 182.0 μg/d, which is above Arth’s threshold of 150 μg/d; therefore, according to the assumption of the Arth’s model, 100% of the preventable NTDs can be prevented by the folic acid fortification program.

All industrially produced flour in Cameroon is subject to mandatory fortification (5), and a negligible percentage of flour is not industrially produced. Therefore, we modified the assumed program coverage from 98% (estimated by Arth et al. using the Food Fortification Initiative database (6)) to 100%. In sum, Arth’s model predicts that 1,936 NTD cases can be averted annually by the current wheat flour fortification program.

1. Wald’s model. Wald’s model w(7) is based on two equations (**eq. 3 and eq. 4)**. First, we used the baseline mean plasma folate concentration (8,9) and the population average additional folic acid consumption from fortified wheat flour based on the CNMS data (4) to predict the plasma folate concentration after fortification by using **equation 3**: original plasma folate concentration + 0.0227 x additional folic acid intake = 18.0 + 0.0227 x 182.0 = 22.1 nmol/L. Then, we used the predicted and baseline plasma folate concentrations in **equation 4** to obtain the percent NTDs averted as

$\% NTD reduction =\left( 1-\left( \frac{new or predicted plasma folate \left( \frac{ng}{mL} \right)}{original plasma folate \left( \frac{ng}{mL} \right)} \right)^{-0.81} \right)\times100\%=\left( 1-\left( \frac{22.1}{18.0} \right)^{-0.81} \right)\times100$ =15.4%

Hence, the number of NTDs averted = 15.4% x [2,334 NTD cases] = 359 NTD cases averted.

1. Crider’s 2019 model. Because plasma folate concentration was measured in two major cities in Cameroon one year after initiation of the national folic acid fortification program, we were able to assess Wald’s (7) and Crider’s 2019 models (10) by comparing their predicted plasma folate concentrations with the observed ones in the two major cities. We calculated the predicted plasma folate concentration based on Wald’s model by using **equation 3**: 14.8 + 0.0227 x 231.5 = 20.1 nmol/L (Wald’s model (7)) and Crider’s 2019 model (10) by using **equation 5**: $baseline serum folate\times{1.116}^{(folic acid intake [\mu g]/100)}=14.8\times{1.116}^{(231.5/100)}=19.1 nmol/L$. These estimates were then interpreted in relation to the proposed plasma folate insufficiency threshold (pf-IT) (11) to assess the adequacy of folate status for reducing NTD risk and compared with the observed plasma folate in two major cities. Unfortunately, we are not able to compare the number of NTD cases averted between the two models, as Crider’s 2019 model (10) only estimates the effect of folic acid intake on plasma folate levels and not on NTD reduction.

**Supplementary references**

1. Walker N, Tam Y, Friberg IK. Overview of the Lives Saved Tool (LiST). BMC Public Health. BioMed Central Ltd; 2013;13:S1.

2. Arth A, Kancherla V, Pachón H, Zimmerman S, Johnson Q, Oakley GP Jr. A 2015 global update on folic acid-preventable spina bifida and anencephaly. Birth Defect Res A. 2016;106:520–9.

3. Tooze JA, Midthune D, Dodd KW, Freedman LS, Krebs-Smith SM, Subar AF, Guenther PM, Carroll RJ, Kipnis V. A new statistical method for estimating the usual intake of episodically consumed foods with application to their distribution. Journal of the American Dietetic Association. 2006;106:1575–87.

4. Engle-Stone R, Ndjebayi AO, Nankap M, Brown KH. Consumption of potentially fortifiable foods by women and young children varies by ecological zone and socio-economic status in Cameroon. Journal of Nutrition. American Society for Nutrition; 2012;142:555–65.

5. Mark HE, Assiene JG, Luo H, Nankap M, Ndjebayi A, Ngnie-Teta I, Tarini A, Pattar A, Killilea DW, Brown KH, et al. Monitoring of the National Oil and Wheat Flour Fortification Program in Cameroon Using a Program Impact Pathway Approach. Curr Dev Nutr. 2019;3:nzz076.

6. Food Fortification Initiative. Global Progress [Internet]. ffinetwork.org. [cited 2016 Nov 22]. Available from: http://www.ffinetwork.org/global_progress/

7. Wald NJ, Law MR, Morris JK, Wald DS. Quantifying the effect of folic acid. The Lancet. 2001;358:2069–73.

8. Shahab-Ferdows S, Engle-Stone R, Hampel D, Ndjebayi AO, Nankap M, Brown KH, Allen LH. Regional, Socioeconomic, and Dietary Risk Factors for Vitamin B-12 Deficiency Differ from Those for Folate Deficiency in Cameroonian Women and Children. Journal of Nutrition. American Society for Nutrition; 2015;:jn210195.

9. Engle-Stone R, Nankap M, Ndjebayi AO, Allen LH, Shahab-Ferdows S, Hampel D, Killilea DW, Gimou M-M, Houghton LA, Friedman A, et al. Iron, Zinc, Folate, and Vitamin B-12 Status Increased among Women and Children in Yaoundé and Douala, Cameroon, 1 Year after Introducing Fortified Wheat Flour. Journal of Nutrition. 2017.

10. Crider K, Devine O, Qi Y, Yeung L, Sekkarie A, Zaganjor I, Wong E, Rose C, Berry R. Systematic Review and Bayesian Meta-analysis of the Dose-response Relationship between Folic Acid Intake and Changes in Blood Folate Concentrations. Nutrients. Multidisciplinary Digital Publishing Institute; 2019;11:71–14.

11. Chen M-Y, Rose CE, Qi YP, Williams JL, Yeung LF, Berry RJ, Hao L, Cannon MJ, Crider KS. Defining the plasma folate concentration associated with the red blood cell folate concentration threshold for optimal neural tube defects prevention: a population-based, randomized trial of folic acid supplementation. American Journal of Clinical Nutrition. Oxford University Press; 2019;145:1636s–10.
